# When did they leave, and why? A retrospective case study of attrition on the Nottingham undergraduate medical course

**DOI:** 10.1186/1472-6920-12-43

**Published:** 2012-06-20

**Authors:** Janet Yates

**Affiliations:** 1 Medical Education Unit, B94 Medical School, Queen’s Medical Centre, University of Nottingham, Nottingham, NG7 2UH, UK

**Keywords:** Medical students, Course attrition, Academic failure, Mental health, Pastoral care

## Abstract

**Background:**

As part of a wider study into students who experience difficulties, we examined the course files of those who had failed to graduate. This was an exploratory, descriptive study investigating how many students left after academic failure or non-academic problems, or simply changed their minds about reading medicine, and at what stage. The aim of the study was to increase our knowledge about the timings of, and reasons for, attrition. This understanding might help to reduce student loss in the future, by informing selection procedures and improving pastoral support at critical times. It might also assist in long-term workforce planning in the NHS.

**Methods:**

Relevant data on admission and course progress were extracted manually from the archived files of students who had failed to graduate from five recent consecutive cohorts (entry in 2000–2004 inclusive), using a customised Access database. Discrete categories of information were supplemented with free text entries.

**Results:**

1188 students registered over the five-year entry period and 73 (6%) failed to graduate. The highest rates of attrition (46/1188, 4%) occurred during the first two years (largely preclinical studies), with 34 students leaving voluntarily, including 11 within the first semester, and 12 having their courses terminated for academic failure. Seventeen left at the end of the third year (Honours course plus early clinical practice) and the remaining ten during the final two clinical years. The reasons for attrition were not always clear-cut and often involved a mixture of academic, personal, social and health factors, especially mental health problems.

**Conclusions:**

The causes of attrition are complex. A small number of students with clear academic failure might require individual educational interventions for remediation. However, this could have substantial resource implications for the Faculty. Mental health problems predominate in late course attrition and may have been undisclosed for some time. The introduction of a structured exit interview may provide further insight, especially for those students who leave suddenly and unexpectedly early in the course.

## Background

Entry into medical school is extremely competitive in the UK. Data from the Universities and Colleges Admission Service (UCAS) shows that 81,422 applications were made for 8000 places in 2010 [1]. During that year, 2500 applications were made to Nottingham and 270 students were admitted. In common with most other medical schools, admission requires high academic standards and evidence of motivation, relevant work experience, attainment outside study, and satisfactory interpersonal skills. Our admissions procedure uses a four-part process: scanning of GCSE results, completion of a computer-marked questionnaire about extracurricular activities and aptitudes, review of the UCAS personal statement against agreed criteria, and a semi-structured interview. In this way we endeavour to rank and select the candidates who are thought to be best suited to the course, and to de-select those thought academically or personally unsuitable. Final acceptance requires good A-levels, normally at least A-A-B at the time of this study.

Medicine is also a costly course, both to the student and to society. Students are committed to full-time study for at least five years, requiring substantial funding for living costs and tuition fees. Public funding via the Higher Education Funding Council for England (HEFCE) and the Service Increment for Teaching (SIFT) contributes over £200,000 per medical graduate (personal communication with Faculty staff).

Students who fail to thrive on the course, or leave before graduation, are therefore a cause for concern. For the individual student there may be substantial financial and emotional costs involved with failure or course withdrawal, perhaps including shame or stigma. For the Faculty, the provision of additional services such as increased pastoral support or resit examinations generates an extra workload. Students who fail drastically but go through the Appeals process, or are called before a Fitness to Practise committee, will also require substantial staff time and resources. And finally, there is a financial cost to society if an expensive medical school place does not lead to graduation. This also has relevance to long-term workforce planning in the NHS, which must take account of the expected number of new medical graduates each year [2,3].

Progression on the course at Nottingham is subject to clearly-defined regulations, and requires minimum academic standards to be reached at all stages. The first two years are primarily preclinical and laboratory sciences, with some patient contact and early professional development. Failed summative examinations may be retaken once at a later date, but further failure leads to an automatic notice of termination. The third year consists of an Honours course (research project plus taught courses), and then the start of the clinical course, all of which must be passed for onward progression. In the later, clinical, parts of the course (Years 4 and 5), progression requires satisfactory completion of clinical attachments as well as the passing of written and practical examinations. Students failing one or more clinical sections may be required to repeat all or part of an attachment or to resit examinations, sometimes after course suspension and re-start. Successful progress also requires professional standards of behaviour to be met at all times; deviations from such standards may invoke Fitness to Practise procedures on behavioural or health grounds [4].

Students may bring forward personal extenuating circumstances, such as acute illness or unexpected family problems, for consideration by the Faculty. If these are accepted, students may re-take their exams as ‘first sits’. Students issued with termination notices have a right to appeal within a set time frame, and against requirements set down by the University. All students have personal tutors plus access to more senior staff, and are advised and encouraged to seek help and advice for anxieties and difficulties, whether they be academic or personal in nature. Meetings with personal tutors are structured and mandatory during the first two years, and available on demand subsequently. Despite these supportive mechanisms, a number of students leave unexpectedly or fail to seek help for their problems until it is too late.

An earlier study showed that 6% of course entrants at Nottingham failed to graduate [5]. As part of a new study into students who did not make satisfactory progress on the course [6], we decided to investigate the files of all those who failed to graduate, from five consecutive cohorts. We aimed to determine how many students left because of academic failure or non-academic difficulties, or simply changed their minds about reading medicine, and at what stage of the course the attrition occurred. We examined the role of health and social problems in students’ failure to complete, and questioned whether timely interventions might reduce attrition.

## Methods

Relevant data on admission and course progress, based on our previous research, were extracted into a customised Access database from the archived files of students who had failed to graduate from five consecutive cohorts (2000–2004 inclusive). Admissions data included age, sex and domicile (defined as UK/EU or Overseas, for Fee purposes), whether school leaver or graduate, and any interview gradings or notes made by the interviewers. In 2000–2003 inclusive, candidates were awarded marks of 0, 1 or 2 against eight criteria; in 2004 this system changed to a grading of A-D for three criteria. Free-text remarks were often but not always made. There were no other major changes to the admissions procedures during the study period.

Course progress data included progression or attrition during each academic year of the 5-year course, adverse health or social factors, examination failures, course suspensions or repeats, termination recommendations and appeals, and voluntary withdrawals.

Discrete categories of information recorded for the study, for example a yes/no option for health problems, were supplemented by free text entries. This type of data category is variable and may be difficult to interpret, but was judged on the basis of whether it appeared to have a measurable or probable impact on the student’s progression. For example, a one-off acute episode of self-limiting illness would not be categorised as a health issue, whereas recurrent migraines leading to missed attendance would be. In this paper, mental health is used as an umbrella term in order to protect confidentiality. It covers a wide variety of disorders including disabling anxiety, eating disorders, and depression of varying severity.

Each student was allotted a confidential Study ID at the time of data entry and no personal identifiers were retained. Data were analysed by running reports from Access or after transferring the file into SPSS v17.

This study was approved by the University of Nottingham Medical School Research Ethics Committee, ref B/11/2009.

## Results

1188 students registered over the five-year entry period and 75 (6%) had failed to graduate by June 2010. Two were still on the course so excluded from further analysis. There were no statistically significant differences in terms of observed socio-demographics at course entry (sex, domicile, age group or declared disability) between those who failed to complete and those who completed despite difficulties [6]. Examination of their A-level grades showed that 63/69 (91%) had at least two A grades and the majority (41/69, 59%) at least three (no data for four students).

**Figure 1:**
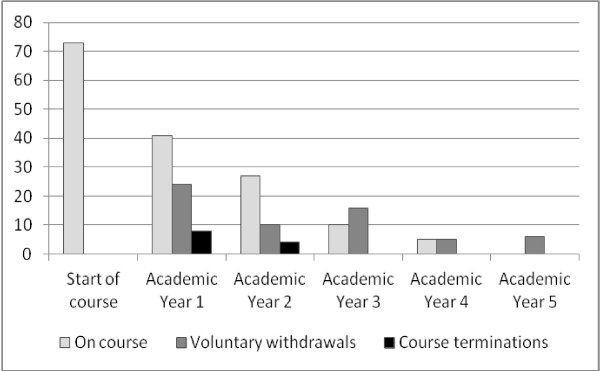
Timing of course attrition during the five academic year divisions, for 73 students who failed to complete the course.

Of the remaining 73, 61 left the course voluntarily and 12 had their courses terminated in Year 1 or 2 for academic failure. The highest rates of attrition were in the early years of the course, as shown in Figure 1.

We examined the patterns and reasons for attrition on a yearly basis. In all cases the year of exit is the academic year of study, regardless of the number of years spent on the course.

### Year 1

There were 24 voluntary withdrawals during Year 1 and eight course terminations for academic failure. Eleven withdrawals were within the first semester, including one who failed to register at all, two who changed their minds about studying medicine within two months, and a third who requested suspension but later withdrew. Six cited personal or family health problems as a reason for withdrawal, and at least 14 transferred to non-medical courses. Reasons for withdrawals, and subsequent career choices, were not always evident in the course files.

Five students repeated the first year, two for health reasons and three after appealing against termination decisions. Of the five, only two proceeded to Year 2, with two withdrawing and one subsequently removed for academic failure. A further three students restarted the course in the following academic year after voluntary suspension, two for personal or family health problems and one who felt too young to be on the course. Two later withdrew and one proceeded after an appeal against termination.

A further seven termination notices were issued after academic failure, three of these students mounting unsuccessful appeals on the basis of family or personal circumstances.

### Year 2

Fourteen students failed to progress beyond Year 2, nine withdrawing and five having their courses terminated. One withdrawal had already repeated Year 1 for academic failure, and another had performed poorly and failed to engage with Faculty support. Three had passed Year 1 but withdrew for personal reasons, one financial and two not happy on the course. Four students withdrew after course suspension, all with health or family problems. The terminations included two students who had been suspended in Semester 3 for academic failure and personal issues, then re-started Year 2. Overall, ten of these students had failed Year 1 exams and at least six appeared to have lost motivation for a medical career.

### Pre-admission features of those who left in years 1 and 2

We reviewed the pre-admission qualifications and interview data for these 46 students who left the course so early. Of the Year 1 leavers, 19/32 (59%) were female, and 5 (16%) domiciled overseas. One was a graduate. The majority of the students (25/32, 78%) had been given normal conditional offers after interviews in December-March; three had unconditional places (1 graduate and 2 overseas students admitted under special arrangements); one was a Reserve offer, and three were late offers made in July-August after earlier rejection. Interview data was missing for four students (two overseas, one graduate, and one late offer). 24 had interview scores, 19 of these being 14/16 or more, and the remainder 12 or 13. Of the four with interview grades, three were AAA and the other ABA.

Eight of the 14 further leavers in Year 2 were female (57%) and two overseas (14%), including one graduate. There was one unconditional offer (graduate) and one late offer, the rest being normal conditional offers. All but one had a score or grade shown, with nine scoring at least 14/16, and three having AAA interview grades.

When the brief comments made at interview were considered, there were few with any notion of concern about the applicant’s commitment, motivation, insight and overall suitability. The only ones noted were “Empathy: didn’t mention listening”; “Empathy: slight self-centred example given”, and ”pleasant, not enormously focused, general insight fair”. The majority were very positive, such as “outstanding”; make offer, very good”; ”motivated, articulate, insight, mature”; “good communicator, team player”.

### Attrition later in the course

Sixteen students left the course at the end of the third year, mostly with good academic records. Four gained a first-class BMedSci degree, two choosing to complete their clinical course elsewhere and two moving to a research career. Of the remaining 12 (nine with an upper second and three a lower second), two withdrew for a non-medical course and one transferred elsewhere for personal reasons. Eight students took suspensions to re-consider their options before withdrawing. Mental health issues affected at least six students who decided to leave the course at this point.

Six students withdrew in the fourth academic year. Four had good academic records and 2.1 degrees, but three of these had long-standing mental health problems and the fourth decided against a medical career. A fifth student had a poorer academic record and finally disclosed health problems and transferred to another course. The sixth had a very poor academic record with disrupted progress and a multitude of non-academic difficulties.

Of even greater concern are five students who withdrew during the final year of the course. Two had good academic records, but succumbed to recurrent mental health problems. Both took time out for treatment but then withdrew. The other three had a history of academic problems in Years 1 and 2. Two eventually failed Finals before withdrawing, and the other withdrew after suspension for health and personal reasons. Two of the three failed to engage with Faculty support.

Interview grades and scores were examined. Of those exiting in Year 3, all had good scores, with positive comments where recorded, with the exception of one. This student was a late interviewee, noted to be “quiet, introverted”, and subsequently suffered mental health difficulties and withdrew.

Amongst the Year 4 leavers, the student with the poorest academic record had been a late interviewee but scored 14/16 and said to be “bright, mature”. One student with mental health problems had a known pre-admission history. Two of the Year 5 withdrawals had been late interviewees, one said to be “very able and pleasant”, the other “bright, articulate, insight, talks well”.

### Other factors affecting students

The data collected suggested that social isolation may also be a significant factor in a small number of students, for example for an overseas student who fails to settle within a peer group, or for any student who lives at home rather than alongside their peers, thereby missing some of the camaraderie of student existence.

There was no numerical evidence that overseas students were more at risk of attrition, but anecdotally we know that a few make very strenuous efforts to stay on the course, sometimes under pressure from family or sponsoring governments. Financial problems may have contributed to students’ difficulties but did not feature in the recorded data. Two late-course leavers disclosed family pressures to read medicine.

### Summary of results

It proved difficult to categorise the reasons for early course exit. In many cases the line between voluntary withdrawal and academic failure was blurred because some students ‘went before they were pushed’, and others transferred out unexpectedly or before sitting exams. However, Table 1 gives a best estimate of the withdrawals over the entire course, and shows the extent of associated health and/or personal issues. Voluntary withdrawals occur throughout the course but especially in the first year, and it seems that some students who realise they have made the wrong decision make an early and positive decision to transfer elsewhere. Withdrawal in the clinical course may be inevitable for students with ongoing health problems despite support and ‘time out’ for recovery. Fear of ‘black-marking’ may have deterred some from seeking earlier help, and external pressure to read medicine may eventually be disclosed as a contributory factor.

**Table 1:** Summary of course attrition during the five academic year divisions, for 73 students who failed to complete the course

**Year/stage of course exit**	Fee Status *	**Voluntary exit/wrong choice of course**		**Academic failure**		**Preference for clinical study elsewhere**	**Reasons for withdrawal not recorded**	**Total**
		**Health or personal problems documented**		**Health or personal problems documented**				
		**Yes**	**No**	**Yes**	**No**			
Year 1								
Semester 1	11 HEU,	2	7	-	-	-	2	11
	2 O/S							
Semester 2	15 HEU,	6	3	3	4	-	-	16
	1 O/S							
Semester 2 after repeat Year 1	3 HEU,	-	1	3	-	-	1	5
	2 O/S							
Year 2								
Semester 3	7 HEU	4	2	^-^	1 ^†^	-	-	7
Semester 4	5 HEU,	1	2	2^**‡**^	2	-	-	7
	2 O/S							
Year 3								
Honours course		-	-	-	-	-	-	0
Clinical Practice 1 (CP 1)	14 HEU,	6	4	-	-	5	1	16
	2 O/S							
Year 4								
Clinical Practice 2 (CP2)	4 HEU,	4	1	1	-	-	-	6
	2 O/S							
Year 5								
Clinical Practice 3 (CP3)	5 HEU	4	1	-	-	-	-	5

* HEU = Home/EU fee status, O/S = Overseas fee status.

† 1 student had already repeated year 1.

‡ 1 student transferred to BSc course.

## Discussion

This study has explored patterns of early course attrition in one medical course over five intakes. Academic failure is a prime cause of early exit in the first part of the course. Recurrent mental health problems, largely anxiety and depression, feature strongly in later course failures. Few of these students gave any cause for concern during their admission interview.

The number of students affected is relatively small, yet still significant for each one who has made the major life decision to read medicine, then either finds it is not the right choice or has to suffer the consequences of course failure. Our best efforts in admission and selection procedures do not always succeed, for a multitude of possible reasons; academic ability at school may not be sufficiently adaptable to meet the demands of the medical course; unanticipated health or social problems may derail progress, or the realities of clinical medicine may be overwhelming. This study has also illustrated that family pressure has, in a few cases, resulted in students who do not have enough personal motivation to thrive and succeed, and perhaps this also was an undeclared factor in some of the students who changed their minds within the first year or two. Currently the Faculty does not ask such students about their decision to leave in any depth, and therefore our information is lacking in detail. There were clearly many interacting factors that we could not analyse. We believe that the introduction of a structured exit interview will help us to quantify these issues in future.

The majority of medical students will have been high-achievers at school and have equally high expectations of their future abilities. They may find it hard to accept that they are not coping academically, and thus be reluctant to seek or accept help even when it is evident to tutors that they need to do so [7]. The students in this study who were given a second chance, by repeating Year 1, yet still failed, may have lacked key strategic learning skills; successful remediation may require very specific interventions to address any deficiencies [8], which has resource implications for the Faculty. A study based on individual student appraisal meetings at another UK medical school has suggested that up to 70% of first year students may recognise the need for advice on study skills, but not all will achieve the necessary goals [9]. Our students are asked about academic issues at their regular meetings with personal tutors, but the extent to which study skills are explored and reflected upon may vary between different tutors and not lead to targeted or mandatory interventions.

Medicine requires physical and mental stamina and resilience [10-12], and health issues were often a significant factor in course attrition. Several students who withdrew in the clinical course had clearly been struggling in the face of poor mental health for some years. Medical students may perceive additional difficulties with ill-health because of fears about confidentiality [13-16] . However, looking after one’s own health and well-being is required by the General Medical Council as a pre-requisite for safe medical practice [17,18].

As yet there is no valid and acceptable way to detect psychologically vulnerable students. Research in Australia has demonstrated the impact of dysfunctional tendencies on academic performance, which may therefore contribute to drop-out [19]. Much attention has been given to the development of valid and robust psychological tests which could be used to screen out candidates with extreme personality characteristics [11]. However, a longitudinal study of students in the UK who sat this test has unfortunately been technically unable to report on any association with drop-outs [20].

Can anything more be done to reduce attrition? In the case of students who leave within weeks after a change of heart, probably not; they had managed to convince our interview panels of their suitability and motivation.

Similarly, there was no conclusive evidence within our data that those who failed to complete the course were less academically able on course entry. A recent systematic review of the literature has concluded that a poorer pre-admission academic record may sometimes be associated with dropout [21], but our students almost all arrive with good academic grades. It will be interesting to see whether the UKCAT test, currently used by Nottingham and many other UK medical schools [22], shows any correlation with course performance or failure. Early evidence is equivocal [23,24]. Measurable academic ability may however be compromised by deficient study processes or external personal and social circumstances, requiring individual support and mandatory remediation [25][26]. Although we strive to offer personal academic support at Nottingham, ultimately it is the student’s responsibility to engage. Those who need the most help may be those who decline [27], and there has to be a line drawn between giving appropriate support and raising expectations too high by ‘failing to fail’ [28].

In the clinical years, when our students are based in a variety of different clinical settings, some distant from Nottingham, tutor contact may be less easy. We are therefore reviewing the support mechanisms for our clinical students.

In the case of severe or recurrent health issues, especially mental health, there is perhaps a need for the GMC to develop additional guidance to medical schools. Current documents state that health concerns can be addressed within Fitness to Practise procedures but do not provide the practical advice that may be required [4]. Although the medical school may seek the advice of Occupational Health physicians when a student has needed ‘time out’ for health reasons, their assurance that a student is fit to continue their studies may still leave doubt about fitness for an on-going career. A small number appear to lack insight into their future ability to function as a doctor, so earlier but more decisive action may be required.

### Limitations of the study

This study has looked at only five cohorts of students at one University, and our data would not necessarily generalise to other medical schools with different entry systems and student profiles. Directly comparable data from other individual UK schools is unavailable, but one study has suggested nearly 5% attrition within the first year alone across all UK medical schools [29], higher than that at Nottingham, where it is essentially unchanged since 1995–1999 [5].

Much of the data collected was textual and relied on notes placed in the student files, and of course on what the students themselves disclosed. It is likely that the incidence of problems is higher than shown, and the reasons for withdrawal more complex. However, a similar mixture of reasons was given by students withdrawing from an Australian course in 1978–89, suggesting that there is a degree of generalisability both over time and internationally [30].

The categorisation of data detailing health or personal issues can be difficult as these issues are idiosyncratic and context-dependent. This categorisation therefore required an element of judgement in some cases. However, the author has no personal knowledge of, or contacts with, any of the students, enabling assessment of data without personal prejudice.

### Future research

Within our own medical school we have a graduate entry stream, with a group of strugglers and non-completers, whose data may reveal different problems; these mature students may have growing families, mortgages, and quite different expectations and pressures from the average 18-year old undergraduate. This part of the study will be reported separately. It would also be interesting to compare attrition on courses at other medical schools, within different intakes and curricula, especially if they conduct and record routine exit interviews. A recent systematic review has revealed the need for more rigorous studies of dropout at medical school [21].

## Conclusions

The causes of attrition are complex. A small number of students with clear academic failure might require individual educational interventions for remediation. However, this could have substantial resource implications for the Faculty. Mental health problems predominate in late course attrition and may have been undisclosed for some time. The introduction of a structured exit interview may provide further insight, especially for those students who leave suddenly and unexpectedly early in the course.

## Competing interests

JY declares no competing interests.

## Author’s contributions

JY is responsible for the design and execution of the study, the analysis and for writing the paper.

## Author’s information

JY has worked as Research Fellow in Medical Education at Nottingham since 2003 and has published a number of papers on student characteristics and progress, mostly in conjunction with David James.

## Pre-publication history

The pre-publication history for this paper can be accessed here:

http://www.biomedcentral.com/1472-6920/12/43/prepub
